# Cezanne predicts progression and adjuvant TACE response in hepatocellular carcinoma

**DOI:** 10.1038/cddis.2017.428

**Published:** 2017-09-07

**Authors:** Jia-hong Wang, Xiao-ping Zhong, Yong-fa Zhang, Xiao-liang Wu, Shao-hua Li, Pei-en Jian, Yi-hong Ling, Ming Shi, Min-shan Chen, Wei Wei, Rong-ping Guo

**Affiliations:** 1 Department of Hepatobilliary Oncology, Sun Yat-sen University Cancer Center; State Key Laboratory of Oncology in South China; Collaborative Innovation Center for Cancer Medicine, Guangzhou, China; 2 Department of Abdominal Surgery, Affiliated Cancer Hospital & Institute of Guangzhou Medical University, Guangzhou, Guangdong, China; 3 Department of Liver Surgery, Fudan University Shanghai Cancer Center, Shanghai, China; 4 Department of Oncology, Shanghai Medical College, Fudan University, Shanghai, China; 5 Department of Laboratory, Fudan University Shanghai Cancer Center, Shanghai, China; 6 Department of Oncology, Guizhou Provincial People’s Hospital, Guiyang, China

## Abstract

We have previously reported that Cezanne could be a prognostic biomarker for survival in hepatocellular carcinoma (HCC) patients. However, the role of Cezanne genes in HCC cells and its response to postoperative adjuvant transcatheter arterial chemoembolization (TACE) in HCC patients remains unknown. In this study, Cezanne expression was detected in human HCC using real-time PCR, western blot and immunohistochemistry. The function of Cezanne in HCC cells was determined by Transwell invasion assays and nude mice metastasis assay. The response of Cezanne in patients who received adjuvant TACE after hepatectomy was evaluated. Functional study demonstrated that interference of Cezanne expression promoted the migration and invasion of HCC cells *in vitro* and boosted metastasized HCC formation in mice. Upregulation of Cezanne diminished the adhesion and migration of hepatoma cells. Further study indicated that Cezanne might inhibit invasion of HCC cells by inducing epithelial–mesenchymal transition (EMT). In addition, patients with low Cezanne expression had significant improvement in prognosis after receiving adjuvant TACE. In contrast, patients with high Cezanne expression had a poorer response to adjuvant TACE. Moreover, Cezanne status was associated with response to adjuvant TACE in patients subgroup stratified by vascular invasion, tumor size and tumor number. In conclusion, Cezanne may be a novel antioncogene that has a pivotal role in the invasion of HCC and contribute to the selection of patients who may benefit from adjuvant TACE to prevent recurrence.

Hepatocellular carcinoma (HCC) is one of the most common solid tumors and prevalent fatal cancers worldwide, especially in East Asia and Sub-Saharan Africa.^1, 2^ HCC generally arises in cirrhotic livers and has a poor prognosis because of the high incidence of disease recurrence after curative treatment.^3, 4^ Therefore, the prevention of recurrence constitutes one of the most important challenges in improving surgical efficacy. Systemic chemotherapy and transcatheter arterial chemoembolization (TACE) are the commonly used adjuvant managements in preventing recurrence and prolonging the survival of patients after hepatectomy. However, although a number of controlled and uncontrolled studies have been performed with most classes of chemotherapeutic agents, no single or combination chemotherapy regimen is significantly effective in HCC,^5, 6^ and only a few of them have shown improved response rates.^7^ Several studies reported that patients with large tumors, venous invasion or intrahepatic metastasis had been recommended to receive TACE 1–2 months after resection.^8, 9, 10^ However, deterioration of liver function after TACE may negatively affect the patients’ prognosis and liver function.^11^ In addition, survival may vary widely among HCC patients with the same clinicopathologic features that is most likely attributable to the heterogeneity of the biological behavior of tumor cells.^12, 13^ Therefore, screening the biomarkers for better valuation of recurrence and prognosis of HCC could revolutionize treatment of HCC and is the key goal of modern personalized medicine.^14^

Cezanne is a member of the A20 family of deubiquitinating enzymes.^15^ Similar to A20, Cezanne has been shown to inhibit NF-*κ*B pathway by deconjugating K63-polyubiquitin chains from RIP-1 and TRAF6,^15, 16^ suggesting that it may have roles in inhibition of cancer progression. Our previous results found that Cezanne was associated with tumor size, vascular invasion and satellite nodule in HCC. Cezanne may act as a potential prognostic biomarker for survival in HCC patients.^17^ Therefore, we hypothesized that the effect of postoperative adjuvant TACE on survival in patients with low Cezanne expression may differ from those with high Cezanne expression. In this study, we evaluated the role of Cezanne genes in HCC and its response to postoperative adjuvant TACE in HCC patients.

## Results

### Cezanne expression is associated with HCC cell invasion

**Figure 1:**
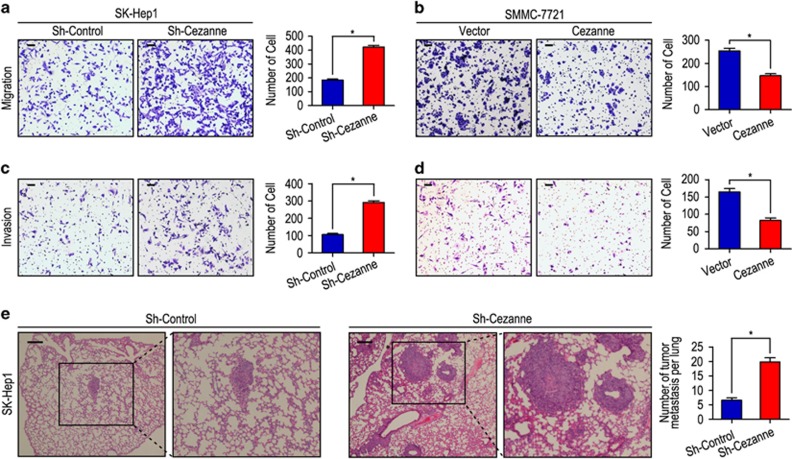
Cezanne inhibits cell migration and invasion in HCC cell lines. (**a** and **c**) Cezanne was downregulated in SK-Hep1 cells and promoted cell migration and invasion. (**b** and **d**) Cezanne was upregulated in SMMC-7721 cells and inhibited cell migration and invasion. (**e**) Lung morphology and H&E staining of nude mice inoculated with SK-Hep1 sh-Cezanne or control cells via tail vein. The number of lung metastatic foci in each group were also calculated. *P<0.05. The scale bar represents 50 *μ*m

Our previous results had reported that Cezanne has a pivotal role in tumor progression and prognosis, and may act as a potential prognostic biomarker for survival in HCC patients. We further investigated the role of Cezanne in tumor cell migration and invasion in HCC cells. Transwell invasion assay revealed that the ability of cell motility and invasion increased in shCezanne-SK-Hep1 cells as compared with those with shCon-SK-Hep1 cells (both *P*<0.001, Figures 1a and c), whereas the ability of cell motility and invasion decreased in Cezanne-SMMC-7721 cells as compared with those with Vector-SMMC-7721 cells (both *P*<0.001, Figures 1b and d). To evaluate the *in vivo* effects of Cezanne on tumor metastasis, two groups of eight mice each were injected intravenously in the tail vein with shCon-SK-Hep1 or shCezanne-SK-Hep1 cells, respectively. After 8 weeks, the mice were killed and the metastatic nodules at the lung surfaces were counted. There were larger numbers of metastatic nodules at the surface of the lungs of mice injected with the shCezanne-SK-Hep1 cells than those with the shCon-SK-Hep1 cells (*P*<0.001, Figure 1e). Hematoxylin and eosin (H&E) staining confirmed that the nodules on the surfaces of mice lungs were metastatic tumors (Figure 1e). CCK-8 assays revealed that the SK-Hep1 with low expression of Cezanne showed more proliferation capacity compared with the control cells. In contrast, overexpression of Cezanne could inhibit the proliferation of SMMC-7721 (Supplementary Figure S1).

**Figure 2:**
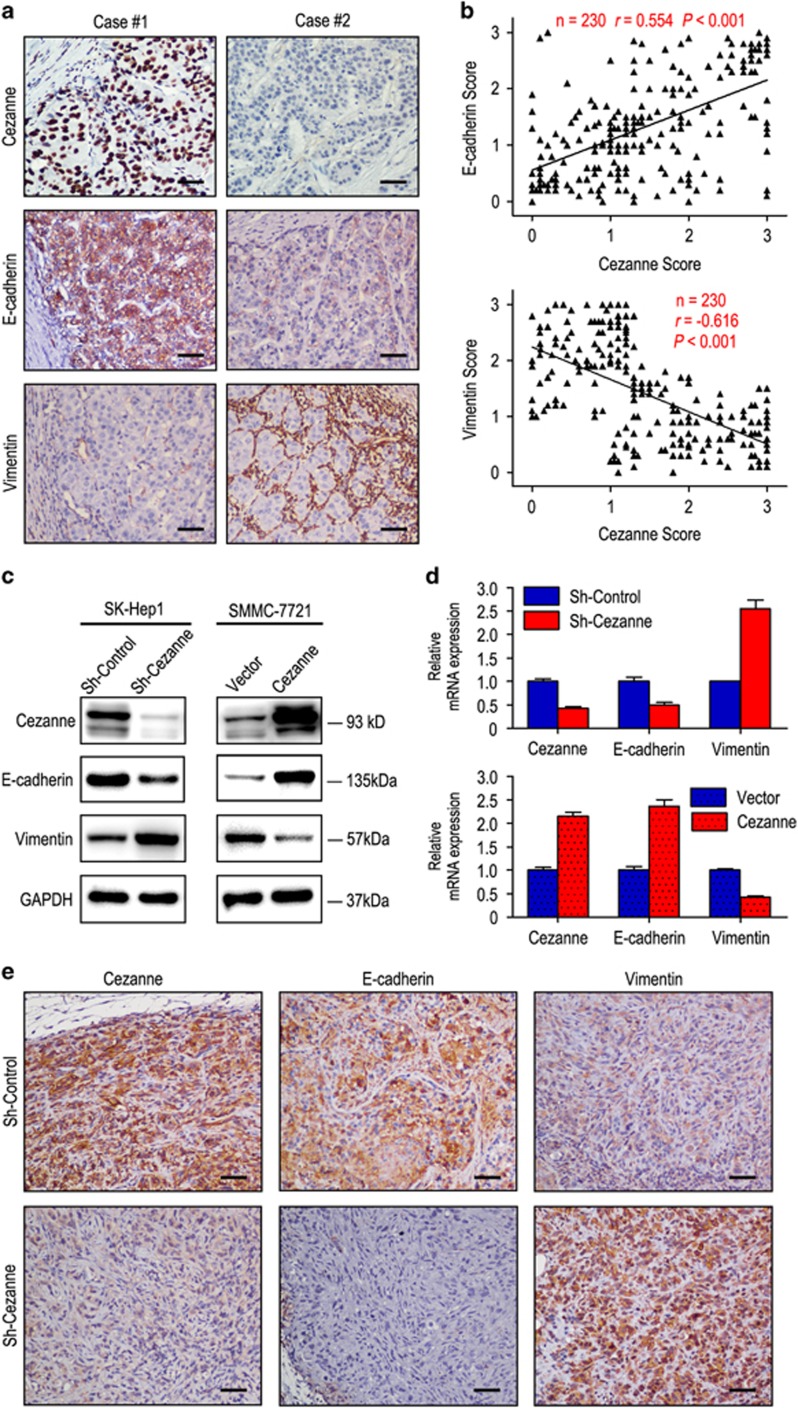
Cezanne expression level correlated with the expression of epithelial–mesenchymal transition (EMT) markers. (**a**) Serial sections of human HCC tissues were subjected to IHC staining with antibodies against Cezanne, E-cadherin and vimentin. In case 1, high expression of Cezanne in HCC tissues was accompanied by elevated E-cadherin and the absence of vimentin. In case 2, low expression of Cezanne was accompanied by the absence of E-cadherin and elevated vimentin. The scale bar represents 50 *μ*m. (**b**) Cezanne expression was positively correlated with E-cadherin expression and negatively associated with vimentin expression. (**c** and **d**) Transfecting siRNAs against Cezanne decreased Cezanne expression, downregulated E-cadherin and upregulated vimentin. Overexpression of Cezanne upregulated E-cadherin and downregulated vimentin. (**e**) Representative images of IHC staining of mice xenografts of SK-Hep1 sh-Cezanne and control cells. The scale bar represents 50 *μ*m

As epithelial–mesenchymal transition (EMT) is one of the key events in tumor invasion and metastasis, the effect of Cezanne on EMT markers was analyzed. The relationship of Cezanne and EMT markers such as E-cadherin and vimentin was confirmed by IHC assays in serial sections of HCC tissues (Figures 2a and b). Moreover, our western blot and RT-PCR results showed that Cezanne expression was positively correlated with E-cadherin and negatively correlated with vimentin expression (Figures 2c and d). Furthermore, decreased expression of E-cadherin and increased expression of vimentin was observed in tumors induced by shCezanne-SK-Hep1 cells compared with those induced by shCon-SK-Hep1 cells (Figure 2e).

### Prognostic significance of postoperative adjuvant TACE within the Cezanne level

**Figure 3:**
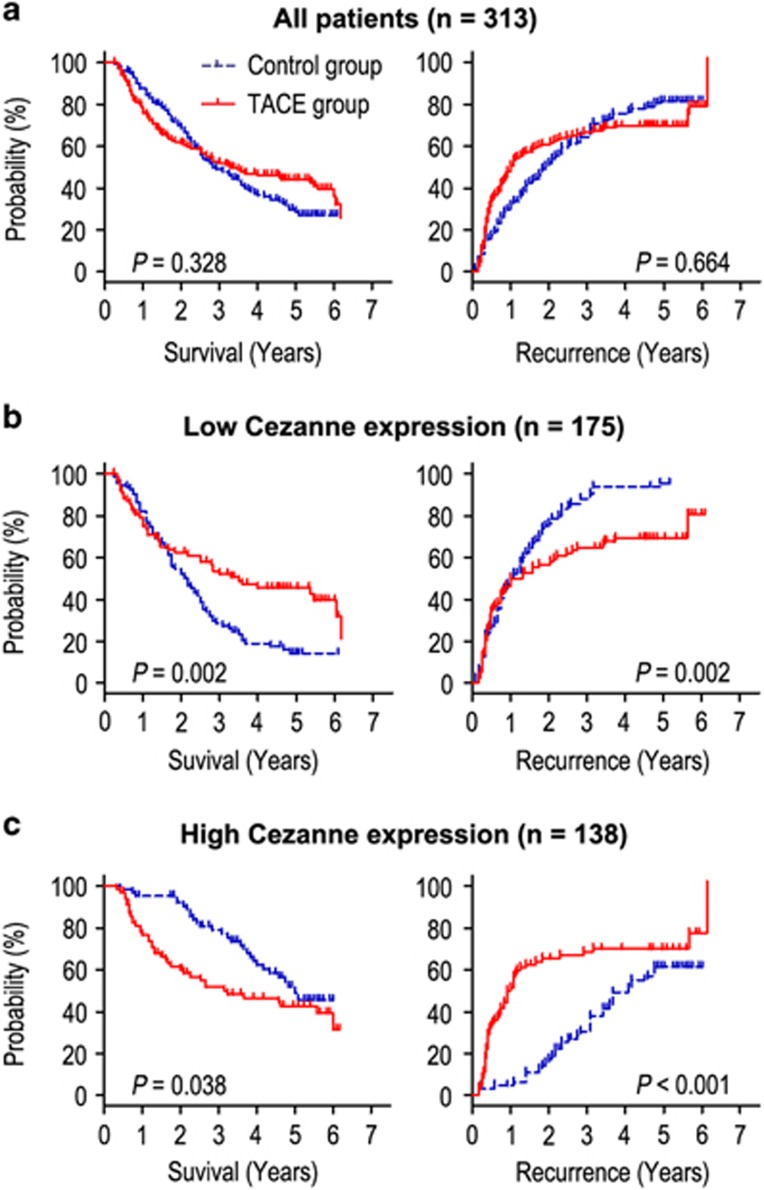
Prognostic significance of postoperative adjuvant TACE according to Cezanne expression. Kaplan–Meier analysis of the correlation between adjuvant TACE therapy and prognosis in all HCC patients (**a**). Kaplan–Meier analysis of the correlation between adjuvant TACE therapy and OS/TTR in patients with low Cezanne expression (**b**) and high Cezanne expression (**c**)

Adjuvant TACE is one of the most used methods to prevent tumor recurrence. However, the curative effect of adjuvant TACE remains unclear. In our results, we found that adjuvant TACE after surgery did not improve the overall survival (OS) and time to recurrence (TTR) rates of the whole study population (Figure 3a). Because Cezanne may predict the prognosis (OS and TTR) of HCC, we next investigated whether Cezanne expression in the tumor was correlated with the response of patients to adjuvant TACE therapy. Patients with low Cezanne expression in tumors had significant improvement in the OS and TTR after receiving adjuvant therapy with postoperative TACE as compared with those without adjuvant TACE (Figure 3b). In contrast, patients with high Cezanne expression in tumors had a poorer response to adjuvant TACE (Figure 3c). The median values of OS times for adjuvant TACE group and Control group were 25.5 and 49.0 months, whereas the median values of TTR times were 11.5 and 41.0 months (Figure 3c). In addition, we also found that the expression of EMT relevant markers (E-cadherin and vimentin) had no correlation with the response of patients to adjuvant TACE therapy (data not show).

In addition, we used Cox proportional hazards regression to further evaluate the correlation between Cezanne expression and response to adjuvant TACE in HCC patients. In the low Cezanne-expressed HCC patients, adjuvant TACE, liver cirrhosis and tumor size were significantly associated with OS (Table 1). Furthermore, Kaplan–Meier analysis demonstrated that HBsAg, liver cirrhosis, lager tumor size and without adjuvant TACE were correlated with shorter TTR in low Cezanne-expressed patients (Table 2). Multivariate Cox regression analysis revealed that adjuvant TACE was an independent predictor for OS (HR=0.536, 95% CI=0.362–0.794, *P*=0.002) and TTR (HR=0.568, 95% CI=0.397–0.812, *P*=0.002) in low Cezanne-expressed patients. However, adjuvant TACE could not act as a potential prognostic biomarker for survival in high Cezanne-expressed patients.

### Cezanne predicts response to adjuvant TACE in clinical subgroups

**Figure 4:**
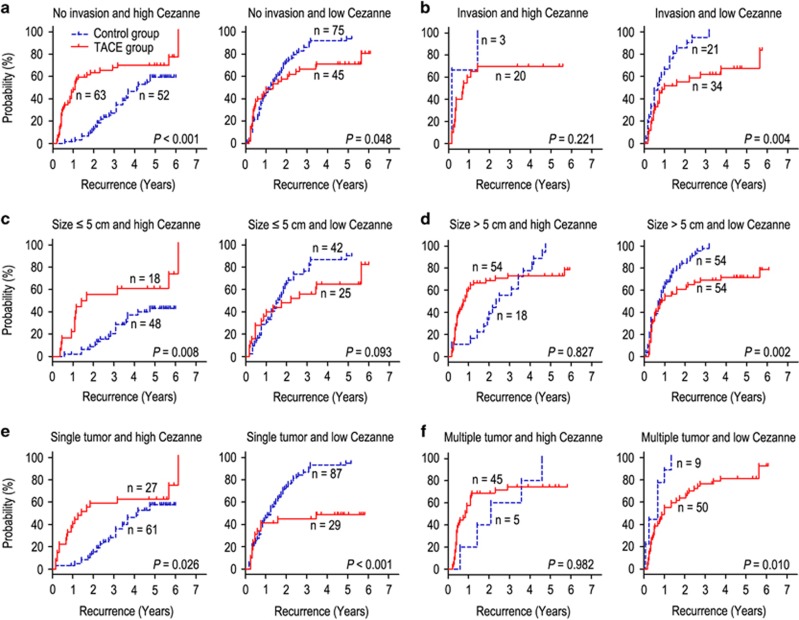
Cezanne predicts response to postoperative TACE in several clinical subgroups. All patients were stratified according to Cezanne levels within vascular invasion (**a** and **b**), tumor size (**c** and **d**) or tumor number status (**e** and **f**). Kaplan–Meier survival estimates and log-rank tests were used to analyze the correlation between adjuvant TACE therapy and time to recurrence in clinical subgroups

The HCC patients with vascular invasion, larger tumor (>5 cm in diameter) or multiple tumors are recommended to receive TACE 1–2 months after resection.^8, 9, 10^ Our findings had similar results with previous study. We found that adjuvant TACE could improve the prognosis (OS and TTR) of HCC patients with vascular invasion (Supplementary Figure S2b) and larger tumor (Supplementary Figure S2d), whereas there were no significant differences in patients with no vascular invasion (Supplementary Figure S2a), small tumor size (Supplementary Figure S2c) and single or multiple tumors (Supplementary Figures S2e and f). Our previous study confirmed that Cezanne could be a potential prognostic biomarker for prognosis in HCC patients. We therefore evaluated the discriminative power of Cezanne levels within the vascular invasion, tumor size or tumor number status on postoperative adjuvant TACE. In patients with no vascular invasion, small tumor or single tumor, high Cezanne expression had a bad response on adjuvant TACE, whereas low Cezanne expression had a better median TTR than control group (Figures 4a, c and e). On the contrary, in the patients with vascular invasion, larger tumor or multiple tumor, high Cezanne expression had no response on adjuvant TACE, whereas adjuvant TACE was associated with significant improvement in TTR of HCC patients with low Cezanne expression (Figures 4b, d and f). Moreover, in the subgroups of no vascular invasion, small tumor or single tumor patients, those with Cezanne overexpression had shorter OS (Supplementary Figure S3a) or no response (Supplementary Figures S3c and d) on adjuvant TACE, whereas those with low expression of Cezanne had a better OS than control group (Supplementary Figures S3a, c and e). In contrast, in the subgroups of vascular invasion, larger tumor or multiple tumor patients, the high Cezanne expression patients had no difference in OS irrespective of whether they received TACE or not, whereas adjuvant TACE could improve the OS in low Cezanne expression patients with vascular invasion or larger tumor (Supplementary Figures S3b, d and f).

## Discussion

Because of high incidence of recurrence and metastasis after hepatectomy, the long survival of HCC patients remains unsatisfactory. In addition, intrahepatic metastasis was thought to have a close relation with the postoperative recurrence.^18^ Adjuvant TACE could inhibit residual tumor after resection of HCC. However, it is also reported that the benefits of adjuvant TACE depended on the selection of patients.^9^ Adjuvant TACE could prolong the survival of patients who had high risks of recurrence in remnant liver because of therapeutic actions on the residual tumor.^19, 20^ Thus, the identification of new predictive biomarkers of HCC invasion and prognosis is critical. In this study, we explored the oncogenicity of Cezanne in the progression of HCC, and analyzed the value of Cezanne in predicting the response of adjuvant TACE in HCC.

Evidences have suggested that NF-*κ*B signaling pathway plays an important role in various liver disease and HCC. NF-*κ*B has been related to initiation, promotion and progression of HCC.^21, 22^ Cezanne expression was shown to be rapidly induced by NF-*κ*B signaling stimulated by TNF-*α* in a negative feedback manner.^16^ In addition, Cezanne negatively modulates NF-*κ*B signaling pathway that has an important role in liver pathology.^16, 23^ Kanki *et al.*^24^ found that low Cezanne mRNA expression was correlated with poor prognosis for HCC patients. Moreover, Cezanne negatively regulated NF-*κ*B signaling pathway that may be an effective target for antitumor therapy for HCC. Our previous results had demonstrated that Cezanne was downregulated in HCC tissues compared with adjacent nontumorous tissues. Moreover, Cezanne could serve as a feasible prognostic biomarker of HCC.^17^ Our functional studies found that short hairpin RNAs (shRNAs) against Cezanne could significantly promote tumor cell proliferation, migration and invasion. These effects were shown to be effectively inhibited when the Cezanne gene was upregulated. The role of Cezanne in inhibiting tumor metastasis was further supported by the *in vivo* experimental metastasis assay. Moreover, our results revealed that Cezanne expression was correlated with the expression of EMT markers. It has been known that EMT has a pivotal role in tumor invasion and metastasis in HCC, and EMT could increase the invasion of tumor cells.^25, 26, 27^ We therefore detected whether the effect of Cezanne on cell invasion was via induction of the EMT pathway. As expected, Cezanne expression led to increased protein and RNA levels of E-cadherin, and decreased expression of vimentin in tissue specimens. Therefore, Cezanne may inhibit HCC cells invasion via NF-*κ*B-mediated EMT pathway, thereby contributing to prolong survival.

Our results found that adjuvant TACE after surgery did not improve the prognosis of the whole study population, a similar conclusion with previous study.^28^ Interestingly, when the patients were stratified according to the expression of Cezanne, the outcomes of therapy with adjuvant TACE were totally different. The patients with low Cezanne expression had a favorable response to adjuvant TACE, whereas patients with high Cezanne expression had adverse response to adjuvant TACE. Moreover, in the patients with low Cezanne expression, adjuvant TACE could be an independent predictor for prognosis. It has been reported that patients with high-risk factors of recurrence, such as larger tumor size or venous invasion, are suggested to receive adjuvant TACE.^19, 20, 29, 30^ However, it was also found that adjuvant TACE after resection had different outcomes of prognosis.^31, 32, 33, 34^ Therefore, combining with molecular biomarkers and clinicopathologic features may help to screen patients who are suggested to receive adjuvant TACE after hepatectomy. Our data revealed that in patients with no vascular invasion, small tumor or single tumor, adjuvant TACE would shorten the TTR in high Cezanne expression patients, and prolong the TTR in low Cezanne expression patients. On the contrary, in the patients with vascular invasion, larger tumor or multiple tumor, adjuvant TACE could not provide benefits in high Cezanne expression patients, and had significant improvement in TTR in low Cezanne expression patients. Similar results were found in OS among different subgroups of HCC patients. These findings suggest that Cezanne status in HCC could be an important indicator in selecting patients who may benefit from adjuvant TACE to prevent relapse. Evaluating the response of adjuvant TACE after hepatectomy, based on the expression of Cezanne and clinicopathologic features, will be necessary for determining whether adjuvant TACE could be used as first-line therapy for HCC patients who have received resection.

It has been known that intrahepatic metastasis is the main reason of early recurrence. The purpose of adjuvant TACE is to kill the metastasis tumor cells in remnant liver. However, it is difficult to estimate whether the remnant liver has minimal intrahepatic metastasis or not after hepatectomy. Our results found that Cezanne was a great predictive factor for prognosis of HCC, and low Cezanne expression predicted vascular invasion and early recurrence in HCC.^17^ Moreover, Cezanne could inhibit the invasion capability of HCC cells. For patients with low Cezanne expression, postoperative adjuvant TACE could decrease the risk of early recurrence and prolong survival. However, TACE may damage remnant liver and deteriorate liver function that is possible to shorten survival of patients with high Cezanne expression who had no metastasis.

## Conclusion

In this study we evaluate the role of Cezanne genes in HCC and its response to postoperative adjuvant TACE in HCC patients. Our results revealed that Cezanne may be a novel antioncogene that has a pivotal role in the invasion of HCC and contribute to the selection of patients who may benefit from adjuvant TACE to prevent recurrence.

## Materials and methods

### Patients and specimens

The study was approved by the institutional review board and human ethics committee of Sun Yat-sen University Cancer Center. Written consent for using the samples for research purposes was obtained from all patients before surgery.

All HCC samples were collected from 313 patients who had undergone curative resections from primary HCC between January 2007 and February 2009 at the Department of Hepatobiliary Oncology, Sun Yat-sen University (Guangzhou, China) (Supplementary Table S1). There were 162 patients who only received curative resections, whereas 151 patients had undergone TACE 1–2 months after resection. The eligibility criteria of the current study were as follows: (1) all the samples were histologically confirmed, (2) none of the patients had distant metastasis or received anticancer therapies before the operation and (3) no serious complications or other malignant diseases. The cases were selected consecutively on the basis of availability of resection tissues and follow-up data. Tumor stage was classified according to the 7th edition of tumor–node–metastasis (TNM) classification of the American Joint Committee on Cancer Staging and the Barcelona Clinic Liver Cancer (BCLC) staging system. OS was defined as the date of liver resection to the date of death or last follow-up. TTR was measured from the date of surgery until the date of relapse, metastasis or last follow-up.

### Cell culture

Two human HCC cell lines were used in this study: SK-Hep1 and SMCC-7721. The two cell lines were obtained from the Liver Cancer Institute of Fudan University (Shanghai, China) and routinely maintained in DMEM supplemented with 10% fetal bovine serum at 37 °C under 5% CO_2_.

### IHC staining

A total of 313 HCC tissues were used in the IHC analysis. Formalin-fixed, paraffin-embedded specimens from consenting patients were cut in 4 *μ*m sections. After being baked at 60 °C for 2 h, the samples were deparaffinized in xylene and rehydrated using a series of graded alcohols. Then, the tissue slides were treated with 3% hydrogen peroxide in methanol for 10 min to exhaust endogenous peroxidase activity. The sections were microwaved for antigen retrieval in 0.01 M sodium citrate buffer (pH 6.0) for 30 min, and then preincubated in 10% normal goat serum for 30 min to prevent nonspecific staining. The sections were incubated with the Cezanne mouse monoclonal antibody (working dilution 1 : 200, Abcam, Cambridge, UK, ab118387), E-cadherin mouse monoclonal antibody (working dilution 1 : 100, Abcam, ab1416) and vimentin mouse monoclonal antibody (working dilution 1 : 200, Abcam, ab8978) overnight at 4 °C. The sections were treated with a non-biotin horseradish peroxidase detection system based on the manufacturer’s instructions (DAKO, Glostrup, Denmark). Assessments of the staining were scored by two experienced pathologists blinded to the patients’ identity and clinical status. In discrepant cases, a pathologist reviewed the cases and reached the consensus.

Both the extent and intensity of immunostaining were taken into consideration when analyzing the data. The intensity of staining was scored from 0 to 3, and the extent of staining was scored from 0 to 100%. The final quantification of each staining was obtained by multiplying the two scores. Cezanne expression was classified as high expression if the score was higher than the median score of 1.3, and if the score was ⩽1.3, the case was classified as low expression.

### Plasmid constructs and transfection

Full-length human Cezanne cDNA was amplified by PCR and cloned into pcDNA3.1(+) expression vector (Invitrogen, Carlsbad, CA, USA), and then transfected into SMMC-7721 cell using Lipofectamine 2000 (Invitrogen) according to the manufacturer’s instructions. Cell transfected with empty vector were used as controls. Stable Cezanne-expressing clones were selected by Geneticin (Rache Diagnostics, Indianapolis, IN, USA) at the concentration of 500 *μ*g/ml.

### Establishment of Cezanne knockdown cells

Lentiviral containing shRNA targeting Cezanne was purchased from Hanbio Biotechnology Co., Ltd (Shanghai, China) and transfected into SK-Hep1 cell using Lipofectamine 2000 (Invitrogen) according to the manufacturer’s instructions. Cells transfected with empty vector were used as controls. Puromycin was used to select stable clones.

### Total RNA extraction and qRT-PCR

Total RNA was extracted from HCC cell lines using TRIzol reagent (Invitrogen) according to the manufacturer’s protocol. The total RNA (2 *μ*g) was reverse transcribed using a PrimeScript RT Kit (Takara, Dalian, China) for first-strand cDNA synthesis. The primer sequences were: Cezanne, forward primer, 5′-TGGCTACCCTGGGGACTTTACTA-3′, reverse primer, 5′-ACTGTCTGGGGAAGGTGGCATA-3′. GAPDH, forward primer, 5′-CTCCTCCTGTTC GACAGTCAGC-3′, reverse primer, 5′-CCCAATACGACCAAATCCGTT-3′. E-cadherin, forward primer, 5′-CGAGAGCTACACGTTCACGG-3′, reverse primer 5′-GGGTGTCGAGG GAAAAATAGG-3′. Vimentin, forward primer, 5′-GACGCCATCAACACCGAGTT-3′, reverse primer, 5′-CTTTGTCGTTGGTTAGCTGGT-3′. The cDNA was subjected to quantitative real-time PCR (qRT-PCR) using the SYBR Green PCR Kit (Applied Biosystems, Carlsbad, CA, USA), and the assay was performed on an ABI PRISM 7900 Sequence Detector. 18S rRNA was used as an internal control. The relative expression level (defined as fold change) of Cezanne (2^−△△Ct^) was normalized to the endogenous 18S rRNA reference (△Ct) and related to the amount of target gene in control sample that was defined as the calibrator at 1.0.

### Western blotting

Western blot analyses were performed according to the standard protocol. The primary antibodies used for western blot are described in IHC method.

### Transwell invasion assays

Invasion assay was performed with BD BioCoat Matrigel Invasion Chambers (Becton Dickinson Labware, Franklin Lakes, NJ, USA) following the manufacturer’s instructions. The matrigel membrane was stained with crystal violet, and migrated cells were counted under a microscope.

### Cell counting kit-8 (CCK-8) assays

Cells were cultured in 96-well plates, in which each hole contained 3000 cells and they were cultured for 24, 48, 72, 96 and 120 h. Cells were allowed for adhesion for a period of 6 h. Subsequently, cells were exposed to 10 *μ*g/ml LPS for different durations. Once the incubation with CCK-8 (Zomanbio, Beijing, China) had been carried out for 3 h, the corresponding optical density (OD) values were detected at 450 nm.

### Experimental metastasis assay

Two groups of 8 mice each were given intravenous tail vein injections of 1 × 10^6^ shControl-SK-Hep1 cells and shCezanne-SK-Hep1 cells, respectively. After 8 weeks, the mice were killed, and the tumor nodules formed on the lung surfaces were counted. The fresh lung samples were harvested and fixed with 10% formalin for histopathology analysis. Tissues were paraffin embedded and sectioned at a thickness of 5 *μ*m. The sections were stained with H&E and then examined under the microscope to count the number of tumor nodules.

### Transcatheter arterial chemoembolization

Adjuvant TACE was performed 1–2 months after hepatectomy. Hepatic arterial angiography was performed first. Among the patients without tumor stain in the remaining liver, preventive chemoembolization was done. The regimen for preventive adjuvant TACE consisted of lobaplatin 50 mg, epirubicin (EPI) 50 mg and lipiodol 5 ml. A contrast-enhanced computed tomography (CT) was performed 1 month later, and the regimen was finished.

### Follow-up

The last follow-up was February 2015. Recurrence was confirmed by serum *α*-fetoprotein (AFP) level, abdominal ultrasonography every 2 months and CT or magnetic resonance imaging or positron emission tomography every 6 months after hepatectomy. The main causes of death were HCC recurrence or complicated cirrhosis of the liver.

### Statistical analysis

The SPSS software package (version 16.0, Chicago, IL, USA) was used for the statistical analysis. The mRNA levels of Cezanne, E-cadherin and vimentin in HCC cells were compared using paired Student’s *t*-test. Pearson’s *χ*^2^ test was applied to analyze the correlation of Cezanne with E-cadherin and vimentin staining scores. Survival curves were generated using the Kaplan–Meier method, and differences between curves were estimated by the log-rank test. All *P-*values were two sided and *P-*values of <0.05 were considered to be statistically significant.

## Publisher’s Note

Springer Nature remains neutral with regard to jurisdictional claims in published maps and institutional affiliations.

## Figures and Tables

**Table 1:** Univariate and multivariate analysis of OS in different Cezanne expressions of HCC patients

**Variables**		**Low Cezanne expression**				**High Cezanne expression**		
	**Univariate**	**Multivariate**			**Univariate**	**Multivariate**		
	***P*****-value**	***P*****-value**	**HR**	**95% CI**	***P*****-value**	***P*****-value**	**HR**	**95% CI**
Gender (female *versus* male)	NS	NS			NS	NS		
Age, years (⩽50 *versus* >50)	NS	NS			NS	NS		
AFP (ng/ml) (⩽400 *versus* >400)	NS	NS			NS	NS		
HBsAg (negative *versus* positive)	NS	NS			0.001	0.003	0.307	0.143–0.662
GGT (U/l) (⩽50 *versus* >50)	NS	NS			<0.001	NS		
Liver cirrhosis (no *versus* yes)	<0.001	0.001	2.504	1.465–4.282	NS	NS		
Tumor size (cm) (⩽5 *versus* >5)	0.014	0.001	1.928	1.310–2.836	<0.001	0.021	1.976	1.108–3.525
Tumor number (single *versus* multiple)	NS	NS			<0.001	0.026	1.894	1.077–3.328
Satellite nodule (no *versus* yes)	NS	NS			NS	NS		
Tumor capsule (no/ incomplete *versus* complete)	NS	NS			NS	NS		
Tumor differentiation (I–II *versus* III–IV)	NS	NS			NS	NS		
Vascular invasion (no *versus* yes)	NS	NS			0.009	NS		
Adjuvant TACE (no *versus* yes)	0.002	0.002	0.536	0.362–0.794	0.038	NS		

Abbreviations: AFP, *α*-fetoprotein; CI, confidential interval; GGT, *γ*-glutamyltransferase; HR, hazard ratio; NS, not significant; OS, overall survival; TACE, transcatheter arterial chemoembolization

**Table 2:** Univariate and multivariate analysis of TTR in different Cezanne expressions of HCC patients

**Variables**		**Low Cezanne expression**				**High Cezanne expression**		
	**Univariate**	**Multivariate**			**Univariate**	**Multivariate**		
	***P*****-value**	***P*****-value**	**HR**	**95% CI**	***P*****-value**	***P*****-value**	**HR**	**95% CI**
Gender (female *versus* male)	NS	NS			NS	NS		
Age, years (⩽50 *versus* >50)	NS	NS			NS	NS		
AFP (ng/ml) (⩽400 *versus* >400)	NS	NS			0.050	NS		
HBsAg (negative *versus* positive)	0.018	NS			0.007	0.019	0.445	0.226–0.874
GGT (U/l) (⩽50 *versus* >50)	NS	NS			<0.001	NS		
Liver cirrhosis (no *versus* yes)	0.001	0.018	1.767	1.104–2.828	NS	NS		
Tumor size (cm) (⩽5 *versus* >5)	0.023	0.001	1.843	1.298–2.617	<0.001	0.005	2.218	1.265–3.899
Tumor number (single *versus* multiple)	NS	NS			<0.001	0.023	2.025	1.103–3.718
Satellite nodule (no *versus* yes)	NS	NS			0.003	NS		
Tumor capsule (no/ incomplete *versus* complete)	NS	NS			NS	NS		
Tumor differentiation (I–II *versus* III–IV)	NS	NS			NS	NS		
Vascular invasion (no *versus* yes)	NS	NS			0.002	NS		
Adjuvant TACE (no *versus* yes)	0.002	0.002	0.568	0.397–0.812	<0.001	NS		

Abbreviations: AFP, *α*-fetoprotein; CI, confidential interval; GGT, *γ*-glutamyltransferase; HR, hazard ratio; NS, not significant; TACE, transcatheter arterial chemoembolization; TTR, time to recurrence
