# Supplementary material for: Cezanne predicts progression and adjuvant TACE response in hepatocellular carcinoma
Source: Cell Death Dis. 2017 Sep 7;8(9):e3043–. doi: 10.1038/cddis.2017.428 (PMC5636974; doi:10.1038/cddis.2017.428)
Supplement: Supplementary Table 1 [file cddis2017428x1.doc]

**Table S1. Patient Characteristics**

| Variable | No. of patients (%) |
| --- | --- |
|
| No. of patients | 313 (100) |
| Age: Median [range], y | 48 [13-80] |
| Gender |  |
| Female | 33 (10.5) |
| Male | 280 (99.5) |
| HBsAg |  |
| Negative | 27 (8.6) |
| Positive | 286 (91.4) |
| AFP: Median [range], ng/mL | 304.4 [0.6-121000] |
| GGT: Median [range], U/l | 58.5 [5.4-655.5] |
| Tumor size: Median [range], cm | 6.0 [1.2-21.0] |
| Liver cirrhosis |  |
| No | 62 (19.8) |
| Yes | 251 (80.2) |
| Child-Pugh class |  |
| A | 308 (98.4) |
| B | 5 (1.6) |
| Tumor number |  |
| Single | 204 (65.2) |
| Multiple | 109 (34.8) |
| Satellite nodule |  |
| No | 223 (71.2) |
| Yes | 90 (28.8) |
| Tumor capsule |  |
| No/incomplete | 216 (69.0) |
| Complete | 97 (31.0) |
| Tumor differentiation |  |
| I | 14 (4.5) |
| II | 177 (56.5) |
| III | 115 (36.7) |
| IV | 7 (2.3) |
| Vascular invasion |  |
| No | 235 (75.1) |
| Yes | 20 (24.9) |
| TNM stage |  |
| I | 145 (46.3) |
| II | 38 (12.1) |
| III | 130 (41.5) |

**Table S1.** Continued

| Variable | No. of patients |
| --- | --- |
|
| BCLC stage |  |
| 0 | 16 (5.1) |
| A | 85 (27.2) |
| B | 139 (44.4) |
| C | 73 (23.3) |
| Adjuvant TACE |  |
| No | 162 (51.7) |
| Yes | 151 (48.3) |
