# Supplementary figures and images for: Cezanne predicts progression and adjuvant TACE response in hepatocellular carcinoma
Source: Cell Death Dis. 2017 Sep 7;8(9):e3043–. doi: 10.1038/cddis.2017.428 (PMC5636974; doi:10.1038/cddis.2017.428)

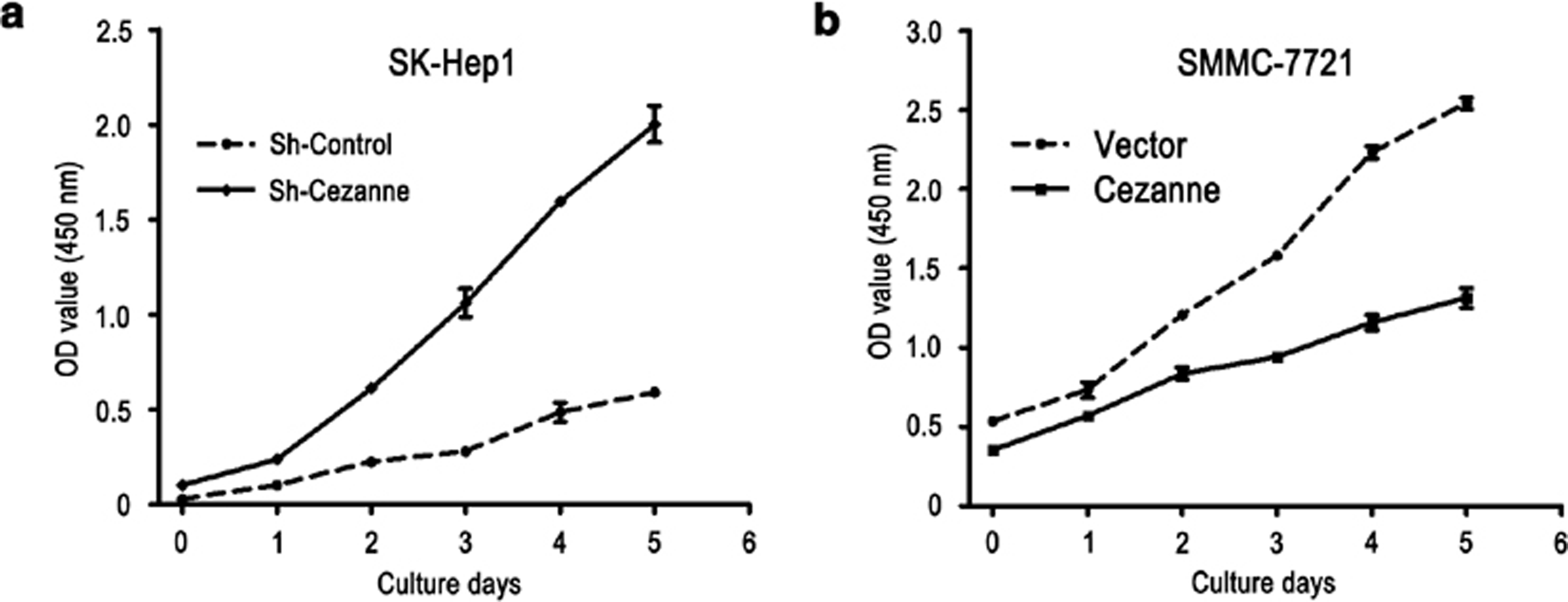

Supplement: Supplementary Figure 1 [file cddis2017428x2.tif]

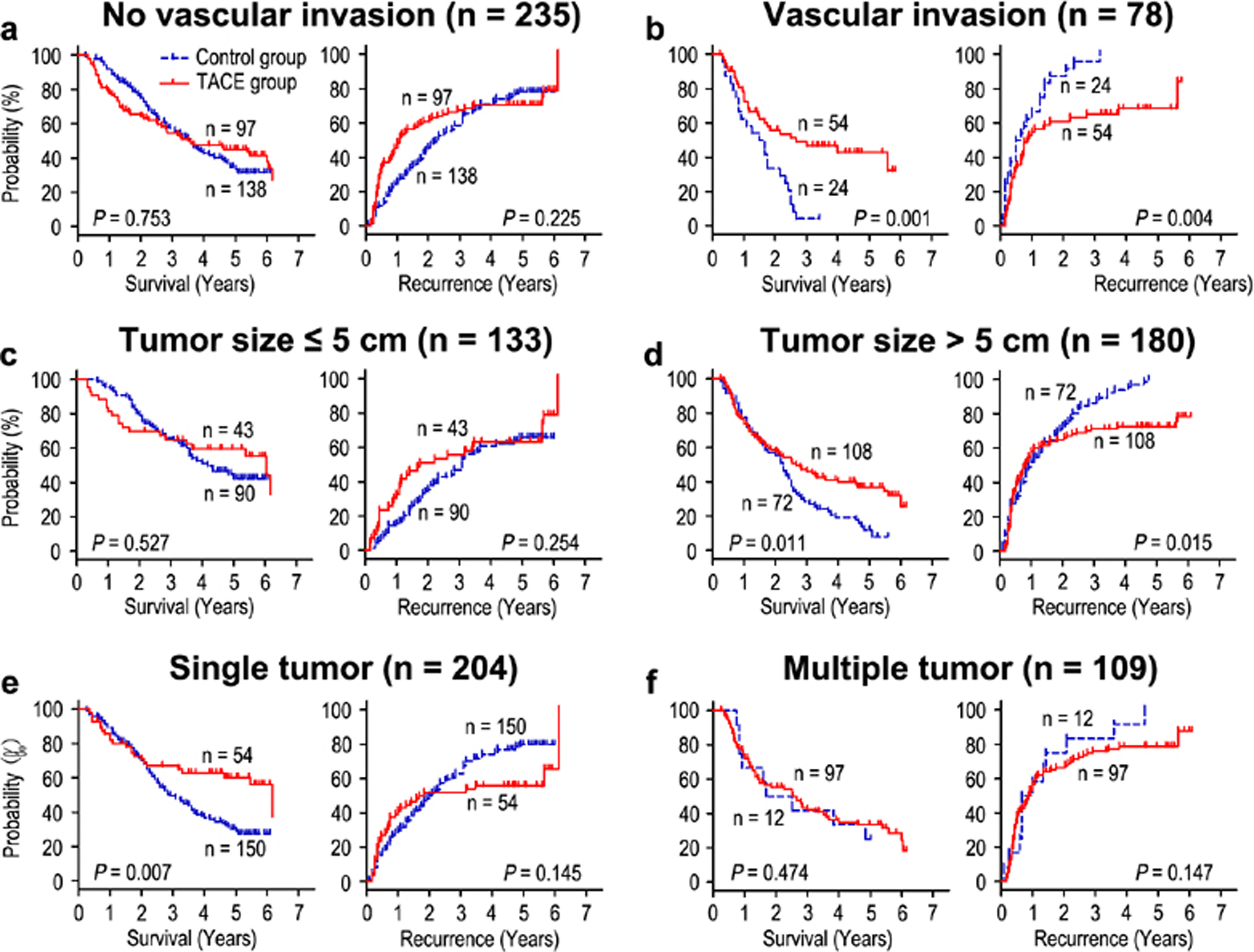

Supplement: Supplementary Figure 2 [file cddis2017428x3.tif]

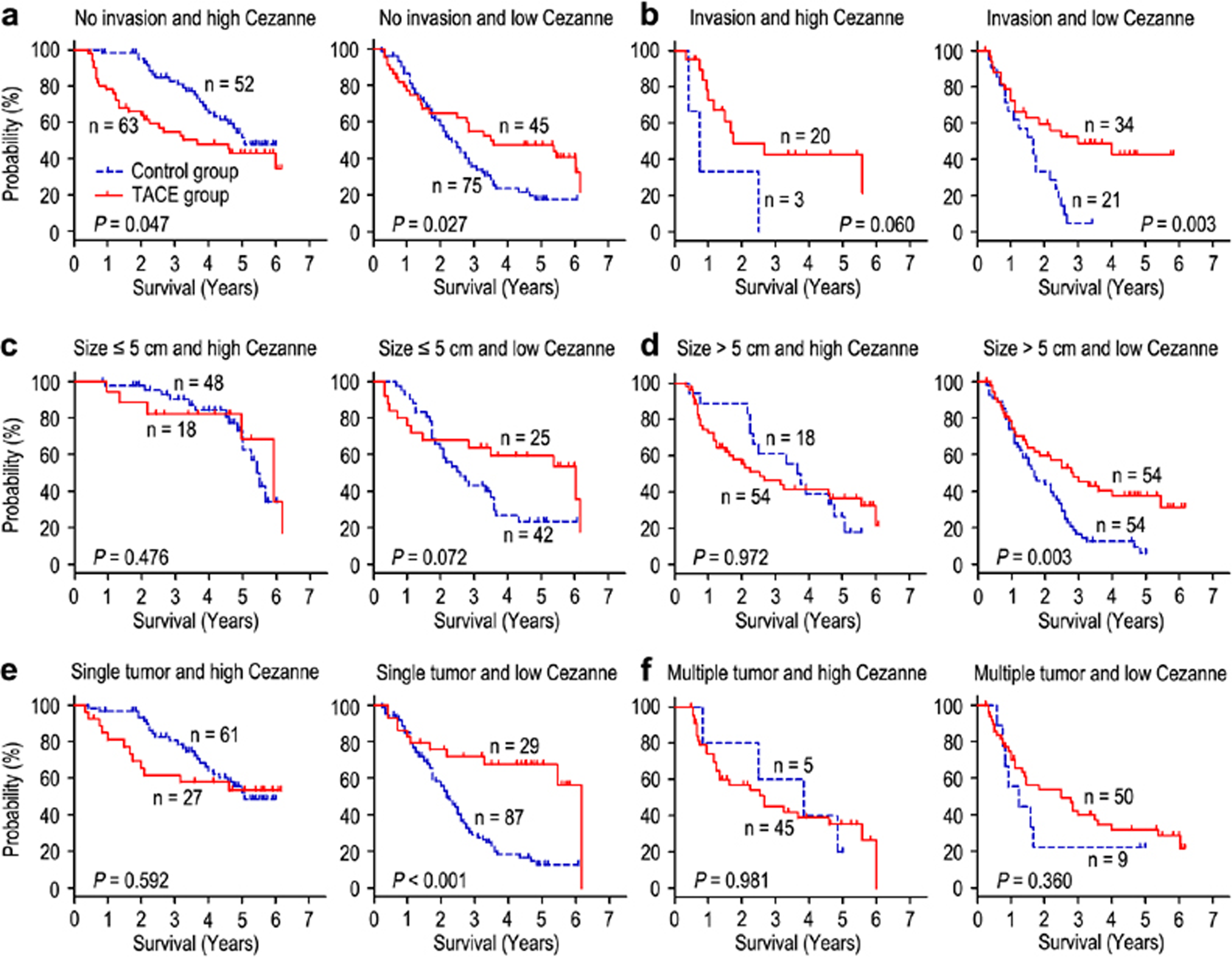

Supplement: Supplementary Figure 3 [file cddis2017428x4.tif]
