# Supplementary material for: Cezanne predicts progression and adjuvant TACE response in hepatocellular carcinoma
Source: Cell Death Dis. 2017 Sep 7;8(9):e3043–. doi: 10.1038/cddis.2017.428 (PMC5636974; doi:10.1038/cddis.2017.428)
Supplement: Supplementary Figure Legends [file cddis2017428x5.docx]

**Supplementary Figure Legends**

**Figure S1.** Prognostic significance of postoperative adjuvant TACE in several clinical subgroups. All patients were stratified according to vascular invasion (a, b), tumor size (c, d) or tumor number status (e, f). Kaplan-Meier survival estimates and log-rank tests were used to analyze the correlation between adjuvant TACE therapy and OS/TTR in clinical subgroups.

**Figure S2.** Cezanne suppressed HCC cells proliferation *in vitro*. Proliferation ability was detected by CCK-8 assay, knock-down of Cezanne promoted SK-Hep1 cells proliferation (a), whereas over-expression of Cezanne inhibited SMMC-7721 cells proliferation (b).

**Figure S3.** Cezanne predicts overall survival to postoperative TACE in several clinical subgroups. All patients were stratified according to Cezanne levels within vascular invasion (a, b), tumor size (c, d) or tumor number status (e, f). Kaplan-Meier survival estimates and log-rank tests were used to analyze the correlation between adjuvant TACE therapy and overall survival in clinical subgroups.
